# Cochlear Implantation Outcomes: A 10-Year Single-Surgeon Experience

**DOI:** 10.7759/cureus.62516

**Published:** 2024-06-17

**Authors:** Abdulkhaliq Emin

**Affiliations:** 1 Otolaryngology, Hawler Medical University, Erbil, IRQ

**Keywords:** prelingual hearing loss, cochlear implantation, rehabilitation, cochlear implant, sir, cap

## Abstract

Background: Cochlear implantation (CI) is an appropriate management strategy for prelingual hearing loss. Rehabilitation programs are essential in enabling children to cope with devices and acquire language skills.

Objectives: This study aimed to assess the hearing outcomes of CIs performed by a single surgeon in Erbil, Kurdistan region of Iraq, and compare them with the results of other CI centers worldwide.

Methods: This is a prospective study implemented in Erbil from November 1, 2013, to October 30, 2023, on a convenience sample of 161 patients with prelingual hearing loss (HL) who underwent CI by a single surgeon. The following were collected: age at implantation, use of hearing aids before implantation, parent's educational level, and duration of rehabilitation. The effect of the previous variables on Categories of Auditory Performance (CAP) score and speech intelligibility rating (SIR) on the auditory levels of children was assessed by the researcher six months and one year following surgery.

Results: Implantation age showed significant associations with the CAP score examined at six months and 12 months post implantation (p-value 0.001). Speech intelligibility rating was also significantly linked to implantation age (p-value 0.001).

## Introduction

Cochlear implantation (CI) is currently a well-established method for restoring hearing to people with profound hearing loss (HL) [1]. Globally, 5% of the population was living with disabling HL in 2018, and is expected to reach 9% of the population by 2050 [2]. It was shown that five infants per 1,000 live births were born with disabling HL, and about 7% of the population with disabling HL are children [3]. In general, the majority of the population with HL is from developing countries, as 2,000 infants with disabling HL are born daily [4]. Hearing loss is generally categorized into prelingual HL (most prevalent) and postlingual HL (less prevalent). Early prelingual HL affects normal children's development, particularly the development of the auditory system, leading to defective development of speech and language [5].

Prelingual HL is a neglected childhood disability characterized by a gradual course that is often painless and physically unnoticed. Early complaints of HL in children might be behavioral problems [6], which are unfortunately subjective and are usually ignored by caregivers [7]. Additionally, prelingual HL leads to many problems for children's lives in the future regarding their physical, psychosocial, and economic status. All these scenarios can be prevented by early detection of prelingual HL through audiological and speech interventions [8]. The existence of a critical period for language development during the first five years of life is well established. Providing auditory stimulation during this period is critical; advanced age at implantation is detrimental to restoring normal auditory processing function. The length of deafness prior to receiving a CI has been reported to be negatively connected with the ability to comprehend and employ verbal-linguistic skills [9].

Many authors have reported the positive effects of cochlear implants on children's language perception skills, speech intelligibility, and voice monitoring. Nevertheless, the CI outcomes are affected by different risk factors, such as intrinsic factors (age at HL onset, intelligence quotient, socioeconomic status, accompanied disabilities) or extrinsic factors (age at CI, educational and re-educational strategies) [10]. 

The common obstacle to rehabilitating children after CI is the delayed development of syntax skills [11], as well as the lower speech intelligibility in children with CI compared to those with normal hearing. For this reason, these children require specialized rehabilitation in the area of speech skills. Another obstacle is that they lack progress in psychosocial development, which requires special programs to help them communicate well with their peers [12, 13].

In Iraq, the cochlear implant program has been started in the last decade, with a gradual rise in the number of implanted cochleae yearly [14].

## Materials and methods

The design of the present study was a prospective study implemented in Erbil in the Kurdistan region of Iraq during the period from November 1, 2013, to October 30, 2023. The studied population was all patients with prelingual HL who underwent cochlear implants during the study duration by one surgeon. The inclusion criteria were prelingual bilateral profound sensorineural deafness in both ears, regardless of age. Exclusion criteria were postlingual HL, inner ear malformation, missing data, and loss of follow-up. The study ethics were implemented in accordance with the Helsinki Declaration with the approval of the Ethical Committee of Hawler Medical University, documented approval of health authorities, and confidentiality of data (approval number: 3347). A convenience sample of 161 patients with HL who had undergone CI was enrolled in the current study after determining eligibility for inclusion and exclusion criteria. All the patients received unilateral CI.

Information about patients was collected by the researcher directly from the parents of children and filled out in a questionnaire designed by the researcher. The questionnaire included the general characteristics of children with CI. The following data were included in the questionnaire: age at implantation, gender, parents' educational levels, period of rehabilitation, duration of device usage during the day, and CI outcomes by Categories of Auditory Performance (CAP) score and Speech Intelligibility Rating (SIR).

The CI surgeries were done by the same surgeon from 2013 until 2023. The implanted devices were the CI Nucleus (Cochlear Corp., Lane Cove, Australia) and Advanced Bionics (Advanced Bionics, Sylmar, CA). We conducted interviews with the parents of the children to collect the scores for CAP and SIR.

The CAP score consisted of eight scores, which were as follows: no awareness of environmental sounds = 0, awareness of environmental sounds = 1, responds to speech sounds = 2, recognizes environmental sounds = 3, discriminates at least two speech sounds = 4, understands common phrases without lip reading = 5, understands conversation without lip reading with a familiar talker = 6, and can use the telephone with a familiar talker = 7. The SIR presents five levels, which are as follows: level 1: coherent speech was not intelligible. Words in spoken language could be recognized only before surgery. The main way of communication was sign language or gestures; level 2: coherent speech was not intelligible, but patient speech could be understood by listeners, mainly through some recognized words combined by context and lip reading; level 3: coherent speech could be understood by listeners focusing their attention with the help of lip reading; level 4: coherent speech could be understood by people with no experience listening to deaf people; and level 5: coherent speech could be understood by everyone. It could be easily understood by children in a daily context. The Cochlear Implant Rehabilitation Program started after the activation of the implant 30 days after the operation.

For the purpose of this study, we categorized the age at insertion of the cochlear implant into four groups: one to three years, four to six years, six to 12 years, and above 12 years. The other group of divisions included parent education, which was divided into six categories according to their level of education: 0 = illiterate, 1 = can read and write, 2 = primary school education, 3 = secondary school education, 4 = high school, and 5 = college. 

Then, we subcategorized the CAP score, which consisted of eight scores, into the other three subdivisions regarding the outcome which were as follows: 0, 1, 2 = poor outcome, 3,4,5 = moderate outcome, and 6,7 = good outcome.

To present the data more clearly, participants were divided into three groups based on their duration of rehabilitation attendance: 0 to six months, six months to 12 months, and 12 months and above.

The patients' information was entered and interpreted statistically by IBM SPSS Statistics software for Windows, version 26 (IBM Corp., Armonk, NY). Suitable statistical tests (Fisher's exact test, independent sample t-test, and one-way ANOVA analysis) for the data were implemented accordingly, with a p-value of ≤0.05 being significant.

## Results

This study involved 161 participants, comprising 52.8% males and 47.2% females, categorized into four groups depending on the age when they received the implant, with more than 80% of participants having undergone implantation operations before the age of seven. Notably, 44.1% of the participants were between the ages of one and three years, while 37.9% were between the ages of four and six. Furthermore, 11.8% were between the ages of seven and 12, with only 6.2% being over the age of 12. The gender distribution was nearly equal among participants, with 52.8% male and 47.2% female. 

All participants in the study had their CAP scores collected at two different time points. The first assessment took place six months after the operation, followed by a second evaluation after a year after the operation (Table 1). Age groups of one to three years and four to six years showed two-point increases in mean scores, whereas age groups of seven and 12 years and >12 years showed one-point increases in CAP scores. Statistically significant variations were noted across all four groups, as shown in Table 2.

**Table 1:** The CAP scores at six months and one year after the operation in each of the implantation age categories CAP: Categories of Auditory Performance

Implantation age	CAP score (at six months)		CAP score (at 12 months)		P*
	Mean	Median	Mean	Median	
1-3 years	2.83	3	5.48	5	<1.000
4-6 years	2.62	3	5.23	5	<1.000
7-12 years	2.21	2	3.79	3	<1.000
> 12 years	2	2	3.45	3	<1.000

**Table 2:** The CAP score categorization at 12 months after the operation in each of the implantation age categories (less and more than six years) CAP: Categories of Auditory Performance

Variables	CAP score at 12 months			Total	P*
	Poor	Moderate	Good		
≤6 Years	24 (18.3)	70 (53.4)	37 (28.2)	131 (100.0)	<0.001*
>6 Years	18 (60.0)	10 (33.3)	2 (6.7)	30 (100.0)	
Total	42 (26.1)	80 (49.7)	39 (24.2)	161 (100.0)	

An assessment was conducted on individuals belonging to the age groups of one to three years and four to six years, measuring their SIR scores at two separate time points which were six months after the operation and one year after the operation. The results of the study revealed a significant rise of one point in the SIR score for both age groups, as presented in Table 3.

**Table 3:** The SIR scores at six months and one year after the operation in each of the implantation age categories SIR: Speech Intelligibility Rating

Implantation age	SIR score (at six months)		SIR score (at 12 months)		P*
	Mean	Median	Mean	Median	
1-3 years	1.24	1.00	2.00	2.41	<1.000
4-6 years	1.13	1.00	2.00	2.21	<1.000

Participants were evaluated on a variety of factors, including their use of hearing aids prior to implantation, the duration of device usage, participation in the rehabilitation program, and the duration of their attendance in the program. Prior to implantation, 33.5% of the patients had used hearing aids. Examination of CI usage patterns revealed that only 6.2% of individuals did not use the CI during a single day, whereas 78.9% used it for more than six hours. Regarding rehabilitation program attendance, 139 participants (86.3%) had participated, with the majority (64.7%) maintaining attendance for a period exceeding six months. The correlation between hearing aid usage before the operation and the CAP score one year post operation was examined (Table 4).

**Table 4:** The CAP score after one year with the use of hearing aids CI: cochlear implantation; CAP: Categories of Auditory Performance

Hearing aid usage before CI	N	Mean CAP after one year	SD	Mean rank	P*
Yes	54	5.11	1.66	83.63	0.605
No	107	5.02	1.82	79.67	

Individuals who had used hearing aids had a mean CAP score of 5.11, whereas those who had not used hearing aids had a mean score of 5.02. The observed difference was not statistically significant. The study examined CAP scores one year post operation in relation to various factors, including parental education, attendance, and duration of rehabilitation programs, as shown in Table 5.

**Table 5:** The CAP score measured after one year in relation to the studied factors CAP: Categories of Auditory Performance

Variables	CAP score (at 12 months)			Total	P*
	Poor	Moderate	Good		
Father's education					
Illiterate	7 (38.9)	8 (44.4)	3 (16.7)	18 (100.0)	
Can read and write	3 (21.4)	7 (50.0)	4 (28.6)	14 (100.0)	0.339**
Primary education	16 (30.8)	21 (40.4)	15 (28.8)	52 (100.0)	
Intermediate level	7 (21.2)	16 (48.5)	10 (30.3)	33 (100.0)	
Secondary education	7 (28.0)	16 (64.0)	2 (8.0)	25 (100.0)	
College	2 (10.5)	12 (63.2)	5 (26.3)	19 (100.0)	
Mother's education					
Illiterate	13 (29.5)	24 (54.5)	7 (15.9)	44 (100.0)	
Can read and write	7 (43.8)	6 (37.5)	3 (18.8)	16 (100.0)	N/A
Primary education	14 (29.2)	16 (33.3)	18 (37.5)	48 (100.0)	
Intermediate level	6 (26.1)	13 (56.5)	4 (17.4)	23 (100.0)	
Secondary education	2 (10.0)	14 (70.0)	4 (20.0)	20 (100.0)	
College	0 (0.0)	7 (70.0)	3 (30.0)	10 (100.0)	
Attending rehabilitation program					
Yes	34 (24.5)	68 (48.9)	37 (26.6)	139 (100.0)	0.169*
No	8 (36.4)	12 (54.5)	2 (9.1)	22 (100.0)	
Period of rehabilitation					
0 to 6 Months	9 (33.3)	13 (48.1)	5 (18.5)	27 (100.0)	0.556*
6 to 12 Months	4 (18.2)	13 (59.1)	5 (22.7)	22 (100.0)	
>12 Months	21 (23.3)	42 (46.7)	27 (30.0)	90 (100.0)	

Regarding fathers' education, individuals with poor, moderate, and good CAP scores were predominantly those with primary school education. For mothers, the highest occurrences were among primary school graduates for those with poor and good CAP scores, while for moderate CAP scorers, the highest frequency was also among primary school graduates; however, no statistically significant relationship was found between parental education and CAP scores. The duration of rehabilitation program attendance was measured in relation to the CAP score, and although no statistically significant correlation was found, the highest percentage of individuals with a good CAP score had participated in the program for more than six months.

## Discussion

In this study, the mean and median CAP scores were recorded in relation to different age groups at different time points which were six months and 12 months after implantation. The results of this study showed significant CAP score variations between the two time points. Also, noticeably, the CAP score outcomes appear to increase with a decrease in implantation age. Those within the age range of one to three years attained an average CAP score of 5.48; the four to six age group achieved 5.23; ages seven to 12 reached 3.79; and those older than 12 reached 3.45. This result correlates with other studies [4,5] that have demonstrated better CAP scores among younger age groups. In fact, in a study done by the Department of Otolaryngology at Mahidol University, it was noted that the age of implantation had an effect on the CAP score. Four out of five children who were under the age of six years old achieved a CAP score of five only 18 months post implantation [15]. The duration of the follow-up could have an impact on the results. In a separate study, it was found that children who underwent implantation before the age of four had a possibility of achieving a CAP score of seven within two years post implantation. However, among those who received implants after the age of four, only 20% attained a normal CAP score of six or seven, and this accomplishment occurred after a considerable postoperative duration. Additionally, approximately only 25% of these children could successfully integrate into the mainstream school system [16]. In our study, we were able to follow up with the patients for up to two years due to a loss of follow-up and the fact that some patients had recently received an implant. This result might be attributed to the critical period around 3.5 years old when the human central auditory system retains its plasticity. Studies indicate that this plasticity persists in some individuals until around the age of seven, after which it gradually diminishes [17].

In addition to CAP scoring, the SIR scoring criteria were also used to evaluate the outcome of CI in this study, in which only age groups one to three years and four to six years were evaluated due to SIR being recommended for use with children up to five years of age, although it can be used for up to 10 years of age. Table 3 demonstrates the mean and median SIR score among the two different age groups recorded at six months and 12 months post implantation. The SIR score increase is statistically significant. However, the progress in each group is slightly different in magnitude; the younger participants show a slightly higher increase in the scores. These data are consistent with other studies that demonstrate the gain of auditory skills and speech perception following CI. We compared both age groups for SIR [18]. In line with this research, other studies indicate swifter advancements in verbal intelligence at younger ages [11,19]. In a retrospective analysis by Alam et al., the SIR of 80 children was assessed at various intervals post CI. Comparing the results, there was a significant statistical difference observed. At three months post operation, the mean SIR score stood at 0.94. However, at six months, it increased to 1.94, followed by 3.66 at 12 months, and further improvement to 4.87 at the 24-month mark [19]. This acceleration could stem from the nervous system's capability to adapt its structure and operations in response to various internal and external needs. Generally, it's widely accepted that the brain is notably receptive to forming new neural pathways during early childhood. Consequently, the earlier children receive implants, the higher the likelihood of improved speech and language development in the future [20]. Another study in India examining cochlear implants across age groups of one to four years and 4.1 to seven years old showed distinct postoperative mean SIR scores at three, six, and 12 months. Although no significant differences were found between the two age groups at three and six months, notable variations emerged at the 12-month mark. In the one- to four-year age range, 37.8% achieved category five post implantation, while only 5.4% in the 4.1-to-seven-year age group reached category five within the 12-month period [21].

Among the 161 participants in this study, one factor influencing cochlear implant outcomes was the engagement and duration of participation in rehabilitation and speech training programs. About 86.3% (139 out of 161) of the participants attended a rehabilitation program. This study shows that the duration of attendance affects the CAP performance score. The highest percentage of participants achieving good CAP scores attended the program for over six months, and the lowest number belonged to the 0 to six months group; the relation was not significant statistically in this study. However, another study also demonstrated that the hearing and speech performance of cochlear implant recipients gradually improved with the extension of rehabilitation time [11]. The duration of the study has also been shown to affect the outcomes. In a study conducted at Zhejiang Chinese Medical University, 80 children who had prelingual deafness and underwent rehabilitation after receiving implants showed that CAP scores improved as the duration of rehabilitation increased, as assessed at one-, three-, six-, and 12-month intervals. Over time, there was a gradual rise in scores, starting from approximately 1.01±2.37 to 4.93±2.68 and 1.41±2.89 to 4.15±2.84, respectively [12].

In this study, 53.7% of the individuals who achieved moderate CAP performance scores had used hearing aids. However, there wasn't a significant contrast found between those who used hearing aids before the surgery and those who didn't. Contradicting these results, another study demonstrated that those who had used hearing aids for three months before CI displayed significantly better scores on the CAP scale [22]. In another study, all the children had used hearing aids for a duration ranging from 2 to 125 months prior to the implantation, based on whether they had any beneficial remaining hearing. Interestingly, a shorter duration of using hearing aids before undergoing CI was linked to better CAP outcomes [15].

Parental education plays a role in categorizing families into different socioeconomic groups, which can influence communication skills in children with normal hearing as well as those with cochlear implants. In this research, the education levels of both fathers and mothers were assessed concerning CAP performance levels. The data showed that among fathers, the highest numbers of poor, moderate, and good outcomes were seen among those who graduated from primary education. While, among mothers, the highest numbers were among the illiterate category with moderate CAP performance scores. No significant relationship was found between educational background and CAP performance. Similar to this, another study also found that the education level of the parents had no effect on the CAP performance [23]. A study conducted in China revealed that parents with higher educational backgrounds frequently enroll their children in auditory and speech therapy programs at rehabilitation centers following cochlear implant surgery. This proactive involvement seemed to positively influence the language development of the children [24].

The limitation of our study is the small number of patients over a 10-year period compared to other studies. This is due to the lack of an insurance system in our locality, which deprives many indicated cases of CI.

## Conclusions

In our cases in the city of Erbil in the Kurdistan region of Iraq, we observed good outcomes regarding auditory and speech perception in children with prelingual hearing loss after cochlear implants and rehabilitation programs. There was a significant correlation between age at implantation and the CAP score and SIR. Although they were not statistically significant, there was a correlation between the CAP score and SIR and the longer duration of rehabilitation, parent education, and use of hearing aids prior to implantation. 

## References

[1] Danasoury IS, Hassan DM, Elkilany AE, Galal EM (2023). Long-term outcome of cochlear implantation on speech perception and quality of life. Egypt J Otolaryngol.

[2] Stevens G, Flaxman S, Brunskill E, Mascarenhas M, Mathers CD, Finucane M (2013). Global and regional hearing impairment prevalence: an analysis of 42 studies in 29 countries. Eur J Public Health.

[3] Bruijnzeel H, Ziylan F, Stegeman I, Topsakal V, Grolman W (2023). A systematic review to define the speech and language benefit of early (<12 months) pediatric cochlear implantation. Audiol Neurootol.

[4] Richter B, Eissele S, Laszig R, Löhle E (2002). Receptive and expressive language skills of 106 children with a minimum of 2 years’ experience in hearing with a cochlear implant. Int J Pediatr Otorhinolaryngol.

[5] Sanju HK, Jain T, Kumar P (2022). Is early cochlear implantation leads to better speech and language outcomes?. Indian J Otolaryngol Head Neck Surg.

[6] Olusanya BO (2007). Addressing the global neglect of childhood hearing impairment in developing countries. PLoS Med.

[7] Ilechukwu GC, Ilechukwu C, Ezeanolue BC (2016). Ear-related problems among children attending the paediatric and otorhinolaryngology out-patients clinics of the University of Nigeria Teaching Hospital, Enugu. Afr Health Sci.

[8] Mulwafu W, Kuper H, Ensink RJ (2016). Prevalence and causes of hearing impairment in Africa. Trop Med Int Health.

[9] Connor CM, Craig HK, Raudenbush SW, Heavner K, Zwolan TA (2006). The age at which young deaf children receive cochlear implants and their vocabulary and speech-production growth: is there an added value for early implantation?. Ear Hear.

[10] Roman S, Rochette F, Triglia JM, Schön D, Bigand E (2016). Auditory training improves auditory performance in cochlear implanted children. Hear Res.

[11] Moradi M, Fallahi-Khoshknab M, Dalvandi A, Farhadi M, Maddah SS, Mohammadi E (2021). Rehabilitation of children with cochlear implant in Iran: a scoping review. Med J Islam Repub Iran.

[12] Xiangyu Q (2019). Effect of cochlear implantation on hearing and speech rehabilitation in pre-lingual deaf children. Front Med Sci Res.

[13] Cook CR, Gresham FM, Kern L, Barreras RB, Thornton S, Crews SD (2008). Social skills training for secondary students with emotional and/or behavioral disorders: a review and analysis of the meta-analytic literature. J Emot Behav Disord.

[14] Alsamady AAA (2019). Effect of cochlear implants in children on parental mental health in Mosul city. Mosul J Nurs.

[15] Thawin C, Kanchanalarp C, Lertsukprasert K, Cheewaruangroj W, Khantapasuantara K, Ruencharoen S (2006). Auditory performance of cochlear implant children aged 2-5 years. J Med Assoc Thai.

[16] Govaerts PJ, De Beukelaer C, Daemers K (2002). Outcome of cochlear implantation at different ages from 0 to 6 years. Otol Neurotol.

[17] Sharma A, Dorman MF, Spahr AJ (2002). A sensitive period for the development of the central auditory system in children with cochlear implants: implications for age of implantation. Ear Hear.

[18] Thabet MT, Said NM (2012). Cortical auditory evoked potential (P1): a potential objective indicator for auditory rehabilitation outcome. Int J Pediatr Otorhinolaryngol.

[19] Alam LCM, Asaduzzaman A, Hossain M, Hossain M, Mahmud T (2021). Effect of age at cochlear implantation on speech and auditory performance. Int J Otorhinolaryngol Head Neck Surg.

[20] Lee KY, van Hasselt CA, Tong MC (2010). Age sensitivity in the acquisition of lexical tone production: evidence from children with profound congenital hearing impairment after cochlear implantation. Ann Otol Rhinol Laryngol.

[21] Shakrawal N, Sonkhya N, Agarwal S, Grover M (2022). The effect of age at cochlear implantation on speech and auditory performances in prelingually deaf children. Indian J Otolaryngol Head Neck Surg.

[22] Jiang F, Alimu D, Qin WZ, Kupper H (2021). Long-term functional outcomes of hearing and speech rehabilitation efficacy among paediatric cochlear implant recipients in Shandong, China. Disabil Rehabil.

[23] Pradhananga R, Thomas JK, Vadivu S, Kameswaran M (2015). Pediatric cochlear implant recipients in India: parental satisfaction with rehabilitation services and correlation with outcomes. J Hear Sci.

[24] Liu S, Wang F, Chen P (2019). Assessment of outcomes of hearing and speech rehabilitation in children with cochlear implantation. J Otol.

